# Electrothermal Behaviour of Constrained Carbon/Epoxy Laminates Under Various Electric Currents

**DOI:** 10.3390/polym18080941

**Published:** 2026-04-12

**Authors:** Gang Zhou, Weiwei Sun

**Affiliations:** 1 Department of Aeronautical and Automotive Engineering, Loughborough University, Loughborough LE11 3TU, UK; 2 C-Power Ltd., Tianjin 300392, China; zhonglizhidian@cpower.com.cn

**Keywords:** carbon/epoxy laminate, electrical conductivity, temperature, clamping torque, thermal conductivity, Lorenz proportionality constant

## Abstract

Although induced Joule heating in carbon/epoxy laminates has been studied, how it can affect their anisotropic electrical conduction has not been well established. The objectives of this work were to ascertain the electrical current–temperature relationship, the effect of induced temperatures on specimen sizes, clamping torques, and electrical conductivity of, and the Lorenz proportionality constants between, thermal and electrical conductivities, all verified with analytical corroborations. A 2-probe method was used in electrical conduction measurements with machined specimens in various dimensions. The specific contributions of elevated temperatures to the electrical conduction through specimen size and clamping torque were ascertained. The thermal conductivities of laminate samples were measured using differential scanning calorimetry. From test results, a parabolic relationship between induced temperature and electrical current was found in both in-plane and through-the-thickness directions. The temperatures in the small specimens rose parabolically. Increasing clamping torques led to linear reductions in temperatures. Over the range of temperatures, the effect of induced temperatures on the electrical conductivity was very small, because the rising of temperatures did not alter the electrical conduction mechanisms. The proportionality constants between thermal and electrical conductivities were established for the first time. This means that just one kind of these measurements needs to be conducted for the same laminates.

## 1. Introduction

The electrical current-induced thermal behaviour in carbon fibre-reinforced laminates occurs in the areas of lightning strike protection [1,2], smart structures [3], electro-thermal de-icing systems [4], electromagnetic interference shielding [5,6], high-speed rotors [7], electrification of composite aircraft [8], and damage detection [9,10]. The common challenges to the uncertain thermal effects faced by these investigations stemmed from the fact that (a) the composite laminates were anisotropic, (b) the lack of measurement standards for electrical conduction for the anisotropic composite laminates led to the random adaptations from those methods for isotropic and homogeneous materials, with a random selection of direct current (DC) levels without much understanding of the current effects on ampere-level electrical conduction, (c) the specific contributions of extrinsic and intrinsic factors to the thermal behaviour were not investigated comprehensively, and (d) there was little info on the analytical relations of raised temperatures to electrical conduction or resistance. This shows that a thorough understanding of the electrical current effects on induced thermal behaviour is essential to exploring the multi-functionalities of lightweight composite laminates in design, analysis, and numerical modelling in the aforementioned areas. It is also a vital initial step to development of the analytical capability of handling the effects of high current-induced thermal damage, pyrolysis, and resin sublimation on composite laminates.

Although multiple endeavours on the current-induced thermal behaviour in carbon/epoxy laminates were reported [1,2,9,11], there is a dearth of knowledge of how either individually or collectively current level, raised temperature, and clamping torque, in addition to conductive paint and fibre waviness, affect the thermal characteristics of these anisotropic carbon/epoxy laminates. Furthermore, for the first time, the correlation of the electrical to the thermal conduction via Lorenz number-like proportional constants for carbon/epoxy laminates is examined. The present systematic experimental investigation focuses on the electro-thermal behaviour of two types of carbon/epoxy laminates under various levels of electrical current and clamping torque. The objectives are to develop a systematic and in-depth understanding of heat elevations within the volume of the specimens and evaluate the effects of both intrinsic and extrinsic factors on electro-thermal behaviour using the two-probe method developed in [12]. While intrinsic factors are fibre waviness, extrinsic factors include current level, raised temperature, specimen size, clamping torque, and conductive paint. The analytical predictions and/or corroborations developed were essential to guide and ensure that the developed data trends were qualitatively correct, due to the pioneering nature of experimental work and the lack of relevant results in the public domain.

## 2. Carbon/Epoxy Laminates Specimens and Preparations

Two types of carbon/epoxy laminates were composed of unidirectional (UD) tape and plain weave fabric prepregs, respectively, in which both used the same 12k PAN-based Grafil 34-700 carbon fibres and LTM45 epoxy resin (from Solvay, Heanor, Derbyshire, UK). The UD tape-based laminate panels of 300 × 300 mm were laid up symmetrically in both 16 and 32 plies, each in a lay-up of UD, cross ply (CP), and quasi-isotropy (QI), with a cured ply thickness of about 0.128 mm. The CP laminates were in a stacking sequence of (0°/90°)_4s_ and (0°/90°)_8s_, whereas the QI laminates were in (45°/0°/–45°/90°)_2s_ and (45°/0°/–45°/90°)_4s_. The fabric-based laminate panel of the same size was laid up with 8 plies in a QI lay-up in (±45°/0°/90°)_2s_, with a cured ply thickness of 0.428 mm. Both types of laminate panels were cured in an autoclave at 60 °C under a pressure of 0.62 MPa for 16 h to have a nominal thickness of 2 and 4 for the tape-based, and of 3.4 mm for the fabric-based laminates, respectively. In LTM45 resin system, epoxide monomer was cured by 4,4′-diaminodiphenylmethane (DDM) hardener. The nominal value of electrical conductivity is 55,556 S/m for Grafil 34-700 carbon fibres [13] and is 1.4 × 10^−12^ S/m for LTM45 epoxy resin [14]. Their respective fibre volume fractions were 61% and 58%.

For the in-plane measurements of electrical conduction, rectangular specimens were used, whereas for the through-the-thickness (TTT) measurements, both rectangular and square specimens were used. The rectangular specimens with a constant width of 10 mm had the respective lengths of 10, 20, 30, 40, and 50 mm. The nominal dimensions of the square specimens were 10 × 10, 20 × 20, 30 × 30, and 40 × 40 mm, respectively. The contact faces of each rectangular and square specimen were machined to parallel on a milling machine. Some selected specimens from both UD tape-based and fabric-based laminates had conductive silver paint applied uniformly on their contact faces.

## 3. Electrical and Thermal Conduction Mechanisms and Relations

### 3.1. Electrical Conduction Mechanisms

At room temperature, a volume electrical conductivity *k* in S/m of typical rectangular specimens made of isotropic and homogeneous materials such as metals is determined by

(1) $$k=\frac{l}{Rbt}=\frac{lI}{VA}=\frac{J}{E}$$

in which *l* denotes the specimen length, *b* the width, *t* the thickness, *R* the resistance in the direction of current flow, *V* the voltage drops between the two electrodes, *A* the cross-sectional area (product of *b* and *t*), *J* the current density, and *E* the field strength. The volume conductivity is the reciprocal of the volume resistivity, and the use of both terms is necessary, as the phrase of contact resistance at the solid electrode–specimen interfaces is conventionally used to address current flow. Equation (1) indicates that the variation of the current density with the field strength should be constant or is linear for isotropic and homogeneous materials.

An electrical conduction mechanism in isotropic and homogeneous materials consists of two parts, the intrinsic *k*_0_ being characterised by electrons interacting with phonons (lattice vibration) and the residual (or extrinsic) *k_i_* being characterised by electrons interacting with chemical and physical imperfections, as represented by:

(2) $$k={{\;k}_{0}\left(T\right)+k}_{i}$$

In the intrinsic part, electrons slow down after the collisions with the crystal lattice structure of a material, even if their average drift velocity increases. Moreover, the collisions cause electrons to scatter and hence increase the material’s resistance to the current flow. The collisions also distort the lattice structure, causing electrons to collide further with other electrons. In the extrinsic part, the interactions with imperfections such as interstices, phase boundaries, impurities, and dislocations scatter electrons so as to decrease the electrical conduction. At elevated temperatures, with more phonons and phonon scattering, the intrinsic part is affected, whereas the extrinsic (residual) part is less so. Thus, increase in temperature leads to decrease in electrical conduction for metals in general, whereas it does the opposite for semiconductive materials, in which the electrical conduction could be of ionic nature.

Continuous carbon fibres-reinforced epoxy (CFRP) laminates are the mixtures of highly conductive carbon fibres and insulative amorphous epoxy with cross-linked microstructure, with carbon fibres making up about 61% of the laminates by volume. They are anisotropic and locally inhomogeneous in any given cross-sectional area. Their electrical conduction mechanisms are completely anisotropic. As carbon fibres with covalent bonds in co-axial basal planes, with the greatest number of charge carriers and highest drift velocity, are oriented only in the in-plane directions of the laminates, both the intrinsic (i.e., electron–phonon interaction) and the extrinsic (electrons–imperfections interaction) parts could exist in these directions, as fibre–epoxy interfaces can be viewed as significant physical imperfections. Thus, it is unclear whether the fibre-dominated electrical conduction in the in-plane fibre directions of CFRP laminates carries mostly the metallic characteristics or not, as current density given by Equation (1) indicates that it is not only position-dependent but also direction-dependent. That is, when temperature increases, the in-plane electrical conduction of the laminates could go either way, though the electrical conductivity of carbon fibres was also reported to increase with an increase in temperature, due to increased electron mobility and/or increased electron jump distance and decreased mean free path [15,16].

In the TTT direction of CFRP laminates, an electrical conduction path network is dominated by either tunnel-through (below glass transition temperature *T_g_*) or hop-over (above *T_g_*) of small epoxy gaps from individual fibre filament to filament. They are again locally inhomogeneous but in any given plane in the TTT direction. The efficiency of tunnelling-through depends on the shape (or height) of charge barriers, filament-to-filament gap distances (varying from nanometre to micron), the availability of current-dependent electrical energy, and the dielectric constant of epoxy. The dielectric constant of epoxy decreases with increase in temperature [17]. There are reports that the elastic tunnelling-through mechanism had energies comparable to those of phonons [18]. Overcoming the barriers, this thermally activated tunnelling-through in terms of local electrical conductivity can be represented by the Arrhenius law [17,19] as

(3) $$k^{l}=k_{0}^{l}exp\left(-\frac{a}{B_{0}T}\right)$$

in which *k^l^* denotes the (average local) electrical conductivity at ambient temperature, *k^l^*_0_ the (local) electrical conductivity at room temperature, *a* the thermally assisted energy (vs energy gaps) of epoxy (for electrons to tunnel through the epoxy gaps between two carbon fibre filaments in the transverse direction), and *B*_0_ the Bolzman constant. In addition, carbon fibres in their transverse (or diameter) direction are formed with van der Waal’s bonds between the basal planes, the number of charge carriers is less than that of their longitudinal directions. Thus, the electrical conduction mechanism characteristics of CFRP laminates in the TTT directions could also be similar to those in the in-plane direction, when temperature is increased. Nevertheless, a key prediction of Equation (3) is that when the conduction mechanism of the material remains unchanged over a range of temperatures, the electrical conductivity of the material is dominated by an asymptote with the magnitude of *k^l^*_0_.

### 3.2. Thermal Conduction Mechanisms

The thermal conduction mechanisms in solid materials depend on their microstructural characteristics in terms of isotropy and homogeneity, in addition to contact conditions. For isotropic and homogeneous metals and other electrically conducting non-metals, thermal conduction has two modes, one being conducted by lattice (atomic and molecular) vibrations about their equilibrium positions *λ_l_* (phonon-based) and the other by the electron-based mobility *λ_e_* via diffusion through the bulk lattice. It can be expressed by Equation (4):

(4) $$\lambda =\lambda_{l}{\;+\;\lambda }_{e}$$

The phonon contributions *λ_l_* come from phonon movement, to achieve thermal equilibium, from high to low temperature regions of a metallic solid, in which temperature gradient exists. The electron movement-based mode dominates thermal conduction in metals, as imperfections such as interstices, phase boundaries, impurities, and dislocations affect the thermal conduction. The electron migration from a high- to low-temperature region contributes to the thermal conduction due to its kinetic energy. Thus, the more electrons available for migration, the greater contribution it makes to thermal conduction. As there are many free electrons in metals, *λ_e_* is much more efficient to the thermal transfer, with electrons moving at higher velocities. As temperature increases, the thermal conduction in metals increases. Nevertheless, imperfections tend to disrupt the heat transfer and cause electron scattering, thereby decreasing the thermal conduction.

For anisotropic CFRP laminates, thermal conduction mechanisms are analogous to those of the electrical conduction in metals in the in-plane directions. As these directions are dominated by carbon fibres, their heat transfer is dominated by lattice vibrations (phonons) and electron mobility. As electrons flowing within a lattice form with strong covalent bonds, they migrate easily through the hot fibre filament regions. Moreover, the transverse direction of carbon fibres is formed with van der Waal’s bonds (between basal planes) so that numerous charge carriers and their higher drift velocities are less than those of the axial direction so that the transverse thermal conduction of carbon fibres is less than that in the axial direction [20]. The thermal conduction in both axial and transverse directions could increase with increase in temperature [16].

In the TTT direction, epoxy has no free electrons, and heat is carried only by phonons. As carbon fibres are the hot regions transversely, thermally inefficient epoxy separating them becomes the cold regions. Thus, it is possible for the interior fibre tows of a laminate specimen to be hotter than the transverse exterior faces until thermal equilibrium is achieved for a given electrical energy. A temperature gradient exists, before attainment of uniform temperature. Thus, in this direction, a thermal conduction of CFRP laminates is conducted primarily by phonons, and its thermal conductivity can be obtained by the Debye equation [21]:

(5) $$\lambda_{l}=\frac{C_{p}vl_{m}}{3}=\frac{c_{p}vl_{m}}{3V}=\frac{{\rho_{ph}C}_{p}vl_{m}}{3}$$

where *C_p_* denotes the specific heat capacity (temperature sensitive), *v* the average phonon velocity (the velocity of sound, temperature insensitive), *l_m_* the average phonon mean free path (temperature insensitive, on an order of the interatomic spacing), and *ρ*_ph_ the phonon density. For thermal conductivity measurements of the prepared specimens, the thermal conductivity of specimens can also be represented by the ratio of the heat flux *q_l_* (or *Q*/*A*) to the temperature gradient (Δ*T*/Δ*l*) as

(6) $$\lambda_{l}=\frac{Q}{A}\frac{∆l}{∆T}=q_{l}\frac{∆l}{∆T}$$

Above can be further manipulated for the thermal conductivity to be related to not only the specific heat capacity but also the density *ρ* of the specimens as

(7) $$\lambda_{l}=\alpha \rho C_{p}$$

in which *α* in unit of m^2^s^−1^ denotes the thermal diffusivity of the specimens.

### 3.3. Wiedemann–Franz–Lorenz Law for Carbon/Epoxy Laminates

In metals, both the electrical and thermal conductivities are dominated by the conduction mechanism of electron mobility. Thus, their conductivities for steady state are linearly related by the Wiedemann–Franz–Lorenz (WFL) law via a Lorenz proportionality constant *L_L_* (or WFL coefficient) over a range of temperatures, as presented by

(8) $$\frac{\lambda }{k}=L_{L}T$$

This means that between electrical and thermal conduction, just one type of these measurements (i.e., the easier one of the two) needs to be performed for a given material with data of the other one being calculated by Equation (8), once the Lorenz proportionality constant becomes available.

For anisotropic CFRP laminates (and other continuous fibre-reinforced laminates), at present, there are simply no Lorenz proportionality constants being published. Neither electrical conduction nor thermal conduction in CFRP is easy to evaluate. For electrical conduction, a properly formulated measurement methodology has just emerged for CFRP, as reported in [12]. Although simple and straightforward for measurements at room temperature (RT) and temperatures moderately above RT, the electrical conductivity values of being either close to *T_g_* or at *T_g_* are difficult to measure, as the specimens could be damaged by Joule heating. For thermal conduction, the use of DSC could just be difficult because typical samples are very small (in milligram) so that it is much more difficult not only to achieve good contact but also to preserve original fibre volume fraction.

In the in-plane direction (i.e., the axial direction of fibres) of CFRP, the electrical conduction is driven by electron–phonon and electron–imperfection interactions, whereas the thermal conduction is dominated by phonons. In addition, there could be electronic contributions to thermal conduction in the axial fibre direction [20]. A Lorenz proportionality constant *L*_L-inp_ for the given CFRP needs to be established. Moreover, thermal conduction in the in-plane directions has greater phonon velocities than that in the transverse (or diametrical) direction so that their corresponding thermal conductivities are significantly greater [20]. In the TTT direction, the electrical conduction is dominated by tunneling-through, whereas the thermal conduction is driven by phonons. A Lorenz proportionality constant *L*_L-TTT_ is needed.

## 4. Measurement Methods

### 4.1. Method and Experimental Set-Up for Electrical Conduction Measurements

**Figure 1:**
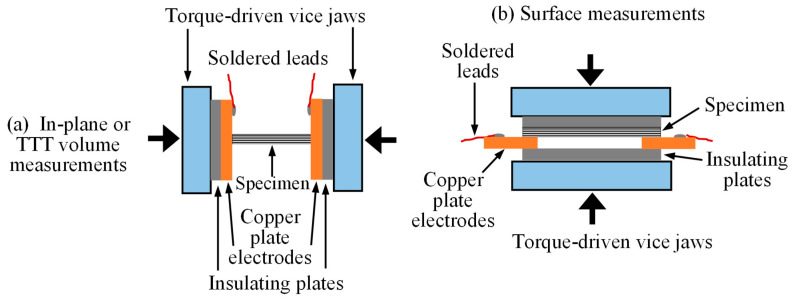
Two-probe method for measurement of volume and surface electrical conductivity.

The 2-probe method (2PM) that was developed in the early investigation [12] requires two solid electrodes to clamp a machined coupon specimen made of a carbon/epoxy specimen. It was developed for correctly measuring not only their volume but also surface electrical conductivity in the same set-up. An experimental arrangement of the method is symbolically shown in Figure 1. Due to the lack of standards for electrical conduction measurement methods for anisotropic composite laminates, the selection of electric current level, clamping torque/pressure value, and conductive paint was required, and the effects of their variations on electrical conductivities, along with the effects of specimen length, thickness, and contact resistance, were comprehensively evaluated [12].

Two digital torque wrenches, with a capacity of 0.3 to 1.5 Nm from screwdriver Norbar 13850 (RS Components, Corby, UK) and 1 to 25 Nm from Wera 7000A (RS Components, UK), were used to regulate torque levels over a wide range through a precision mechanical vice. A range of electrical currents from 0.5 to 3 A supplied via a digital multimeter (Fluke 8050A model, from RC Components, UK) were used to examine the effect of current levels on raised temperatures and eventually electrical conduction. While the specified current range was suited to the middle (20 mm and 30 mm long) and large (40 mm and 50 mm long) specimens, the level of 2.5 A was found to be the upper current limit in trials and errors to the smallest specimens (10 mm long) when substantial Joule heating damage was found with smoke and smell. An IR dual-laser non-contact thermometer RS820 (RS Components, UK) with a digital display was used for rapid precise temperature measurements in a range of −50 to 380 °C with an accuracy of ±1%. Surface temperature readings at the middle of a side surface of the specimens were taken at approximately one minute, by when approximate isothermal state was assumed to be established. There was no guarantee that temperatures within the interior of the specimens would reach thermal equilibrium. It was also assumed that the radiation losses from the specimen faces throughout the period of current supply were negligible. This non-contact laser technique did provide the advantages over thermocouples in the TTT temperature measurements, where the TTT space was very tight. Other techniques of temperature measurements such as thermocouples [2,8] and ovens [11] were also used to measure surface temperatures.

### 4.2. Electrical Resistance–Temperature Relation for Clamped Carbon/Epoxy Laminates

For a clamped laminate specimen set up in an electrical conduction measurement, the electrical resistance throughout its volume consists of two contributions, the temperature-dependent resistance changes and the geometric changes due to thermal expansion, as

(9) $$R=R_{0}\left[1+\alpha \left(T-T_{0}\right)-\varepsilon_{c}\left(1+\left({\nu_{21}+\nu }_{12}\right)\right)\right]=R_{0}\left[1+\alpha \left(T-T_{0}\right)-\frac{P}{E_{c}}\left(1+\left({\nu_{21}+\nu }_{12}\right)\right)\right]$$

in which *R*_0_ denotes the resistance measured with 10 mA at RT *T*_0_, *α* temperature resistance coefficient in per degree, *T* raised temperature, *P* clamping pressure converted from an applied clamping torque, *E_c_* either fibre-direction compressive modulus in the in-plane measurements or compressive modulus of epoxy in the TTT measurements, *ν*_12_ and *ν*_21_ their major and minor Poisson’s ratios. Data on the electrical resistance temperature coefficient (ERTC) α of carbon/epoxy laminates are very scarce. For the temperature range of 20 °C to 60 °C, the unloaded carbon/epoxy laminates were found to show the negative ERTC value in both the in-plane direction [22] and the TTT direction [11] so that the resistance fell when temperature increased [11,22] though the positive ERTC value in the in-plane direction was also reported in [23].

With both temperature and axial compression being considered, the volume electrical conductivity *k* is given as

(10) $$k=\frac{\frac{l}{btR_{0}}}{\alpha \left(T-T_{0}\right)+1-\frac{P}{E_{c}}\left[1+\left({\nu_{21}+\nu }_{12}\right)\right]}\;$$

This shows that the conductivity is parabolically dependent on temperature, though the resistivity is linearly dependent on temperature.

Application of electric current leads to a significant temperature gradient across the laminate specimen in the direction of current flow initially, and, over time, the temperature could approach steady state and uniformity (below *T_g_*). Raising the current further up to the ampere levels within the volume of the small coupon specimens could induce significant Joule heating. As thermal energy is time-dependent, heat *Q* in Joules in the clamped specimens over time *τ* (in seconds) was obtained from

(11) $$Q=I^{2}\tau R={I^{2}\tau R}_{0}\left[1+\alpha ∆T-\frac{P}{E_{c}}\left(1+\left({\nu_{21}+\nu }_{12}\right)\right)\right]=mC_{p}∆T$$

Equation (11) in the split form between the temperature-dependent resistance and the thermal expansion-dependent geometric change, indicates theoretically that increase in current could result in a parabolic increase in raised temperature, which was demonstrated experimentally in [22]. In the present work, Joule heat over a period was assumed to be stored in the specimen as its kinetic energy to raise the specimen temperature without radiation losses.

### 4.3. Method and Experimental Set-Up for Thermal Conduction Measurements

For thermal conductivity measurements, laminate rectangular block samples were cut from the 4 mm thick QI carbon/epoxy panels in both in-plane and TTT directions, and cylindrical disk samples were drilled in the TTT direction from the same panels. Typical weights of samples were in the range of 30 mg up to 90 mg. They were cleaned, dried up, and kept in a desiccator before testing. TA Instrument Q2000 DSC with a refrigerated cooling system 90 (RCS90) attachment was used to conduct experiments under temperature-moderated DSC (MTDSC) using both ramping and quasi-isothermal methods. In the latter method, the modulation period and the modulation amplitude were 80 s at ±0.5 °C, with 10 °C increment and 10 min isothermal hold for a total of nine increments. Glass transition temperature *T_g_* of these carbon/epoxy laminates was about 100 °C.

**Figure 2:**
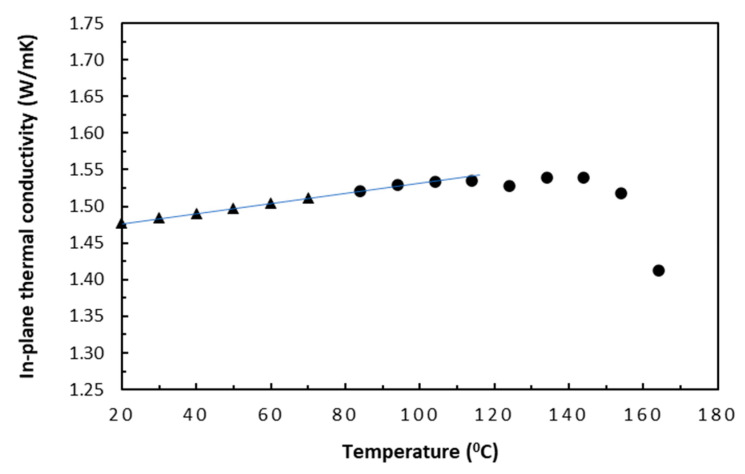
In-plane thermal conductivities of tape-based laminates.

**Figure 3:**
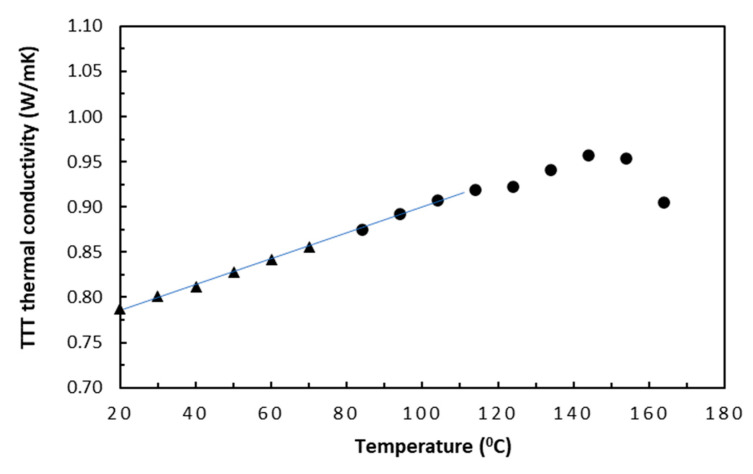
TTT thermal conductivities of tape-based laminates.

Although thermal conductivity test data for both tape- and fabric-based laminates were available, only the tape-based data were presented here in Figure 2 and Figure 3, for both the in-plane and TTT directions. To obtain reliable data trends, the selected nominal start and finish temperatures were 20 °C and 160 °C, respectively, with an increment of 10 °C. This nominal upper cut-off temperature of 160°C was conservatively estimated using independent thermogravimetric analysis (TGA) test data of the same laminate samples. With samples of about 35 mg being tested in air, test data indicated that about 1% mass loss that was attributed to dehydration occurred at about 190 °C, which could signal the incipient damage, and that a sublimation start temperature of LTM45 epoxy was found to be around 225 °C. Thus, the choice of 160 °C ought to be well clear of the potential risks for inducing irreversible thermal damage in the carbon/epoxy samples.

As shown in Figure 2, a steady linear rising trend of thermal conductivity from 1.46 to 1.53 W/mK (about a 5% increase) could be formed from data from 20 °C to about 100 °C (*T_g_* for epoxy) for the in-plane direction. The thermally efficient carbon fibres of about 61% volume in this direction dominated the thermal conduction with their increased electron mobility, whereas the increased mobility of molecular segments in epoxy increased phonon mean free path [21], as temperature increased. This was in accordance with the fact that the small coefficient (about 0.875 × 10^−3^) for the thermal conductivity–temperature relationship in this region is positive. This characteristic could be represented by Equation (12) for the in-plane direction, with *λ*_0_ and *α_T_* being obtained through a curve fitting. From 100 °C or the *T_g_* of epoxy to about 142 °C, the overall trend of thermal conductivity pretty much levelled off. From Equation (7), this seemed to suggest that its thermal energy was absorbed to promote a characteristic jump in specific heat capacity [24,25] in the endothermic transition region. Over this region, PAN-based carbon fibres (T300), which are very similar to the present PAN-based Grafil 34-700, could increase linearly up to 200 °C [26]. Therefore, this also suggested that, in the in-plane direction, epoxy could make an even greater contribution to the thermal conduction of the carbon/epoxy samples than carbon fibres. After 142 °C, it started decreasing due to the significantly increased phonon scattering associated with increased lattice vibrations in epoxy, which hindered the mobility of phonons and reduced their ability to conduct heat. Thus, the conductivity–temperature coefficient became negative [21].

(12) $$\lambda_{l}=\lambda_{0}\left[1+\alpha_{T}\left(T-T_{0}\right)\right]=1.475\left[1+0.000875\left(T-T_{0}\right)\right]$$

In the TTT direction, where epoxy and carbon fibres were in a sequential arrangement, data in Figure 3 showed a similar linear trend from 0.785 to 0.920 W/mK (17%) up to about 100 °C, *T_g_*. The conductivity–temperature coefficient in this direction is about 1.688 × 10^−3^, which is nearly twice the in-plane direction value. Again, such characteristic could be represented by Equation (13) for the TTT direction, with *λ*_0_ and *α_T_* being obtained through a curve fitting. After slight stagnation after 100 °C (i.e., around 120 °C), a significant linear increase of conductivity from 0.92 W/mK to 0.955 W/mK (22%) in the ‘rubbery’ state of epoxy occurred and continued up to 145 °C with the increased molecular motion. This could be because increased molecular motion and segment mobility in epoxy allowed the molecular chains to absorb more thermal energy, leading to a higher specific heat capacity compared to the ‘glassy’ state. Towards the end of this transitional region, a noticeable initiation of density reduction was observed in [27,28], in addition to potential epoxy degradation in [29], and the reduced temperature gradient in the degradation of epoxy [21]. With further increase in temperature, thermal conduction, again, decreased due to the significantly increased phonon scattering, especially at fibre–epoxy interfaces [30], which was accompanied by a negative conductivity–temperature coefficient [21]. In this TTT direction, the less-efficient epoxy dominated the thermal conduction of the laminate samples even more [31].

(13) $$\lambda_{l}=\lambda_{0}\left[1+\alpha_{T}\left(T-T_{0}\right)\right]=0.785\left[1+0.001688\left(T-T_{0}\right)\right]$$

## 5. Results and Discussion

### 5.1. Effect of Electrical Current on Raised Temperatures

**Figure 4:**
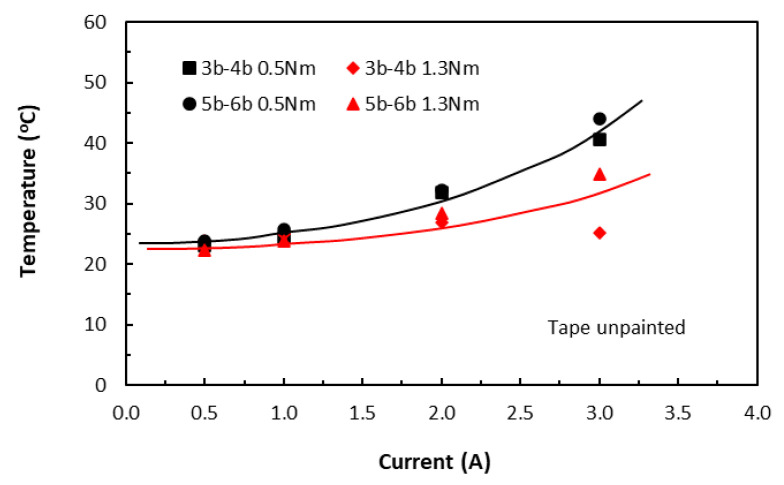
Variations of raised temperature with current on the laminate surfaces of unpainted tape-based in-plane specimens. (3b–4b for 20 mm length and 5b–6b for 30 mm length specimen groups. 0.5 Nm and 1.3 Nm for clamping torque levels).

The variations of induced temperatures from increased electrical current in the in-plane direction of the tape-based laminate specimens were shown in Figure 4. The ascending parabolic trends of temperatures induced by the current levels of 0.5 to 3 A were established for the 20 mm (3b–4b group) and 30 mm (5b–6b group) long unpainted specimens, respectively. However, for the short or small coupon specimens (i.e., 10 mm long), the use of a current level of 2.5 A to 3 A led to their destruction, with temperatures racing through 100 °C (*T_g_*) in a minute. Temperatures in interior carbon fibres were expected to increase with the increased current levels due to Joule heating and thus epoxy separating them was also heated. The rate of temperature rise in epoxy was significantly slower than that of carbon fibres, leading to the nonlinear parabolic trend. This trend agrees with the prediction of Equation (14) below, which was obtained through manipulations of Equation (11).

(14) $$∆T=\frac{R_{0}\tau \left[1-\frac{P}{E_{c}}\left(1+{\nu_{21}+\nu }_{12}\right)\right]I^{2}}{mC_{p}-R_{0}\tau {\alpha I}^{2}}\;\;\;\;for\;0<I<3\;A$$

**Figure 5:**
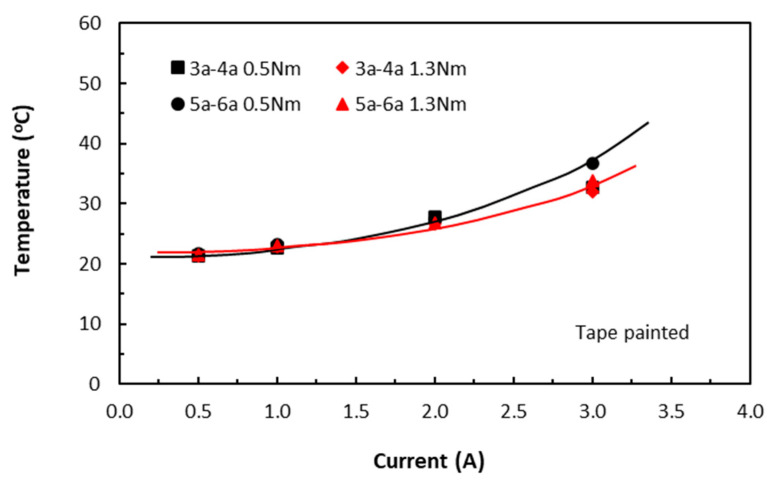
Variations of raised temperature with current on the laminate surfaces of painted tape-based in-plane specimens. (3a–4a for 20 mm length and 5a–6a for 30 mm length specimen groups. 0.5 Nm and 1.3 Nm for clamping torque levels).

The similar specimens with contact faces being applied with conductive paint showed much the same response, as shown in Figure 5. Similar trends were also reported in [8,22]. This also confirmed that the nonlinear parabolic trend of the induced temperatures was independent of the presence of conductive paint at contact faces. Moreover, the temperature levels in the painted specimens were noticeably slightly less than those of the corresponding unpainted specimens. This could be because conductive paint on the contact faces may have dissipated some heat, which led to lower body temperatures. Again, this trend could be represented by Equation (14).

**Figure 6:**
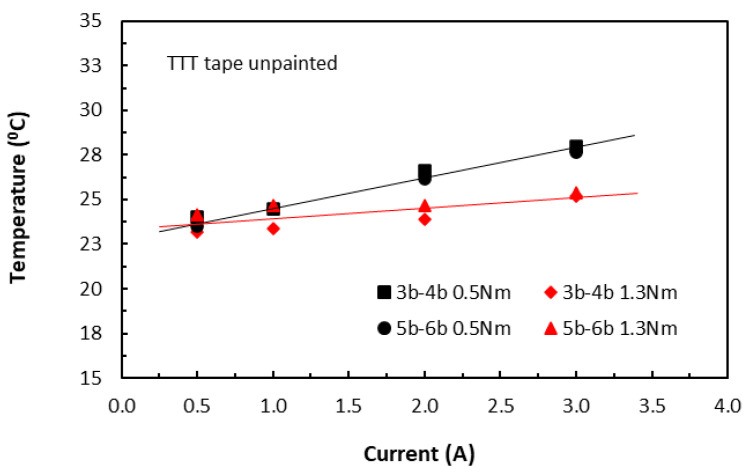
Variations of raised temperature with current on the surfaces of unpainted TTT specimens. (3b–4b for 20 mm length and 5b–6b for 30 mm length specimen groups. 0.5 Nm and 1.3 Nm for clamping torque levels).

**Figure 7:**
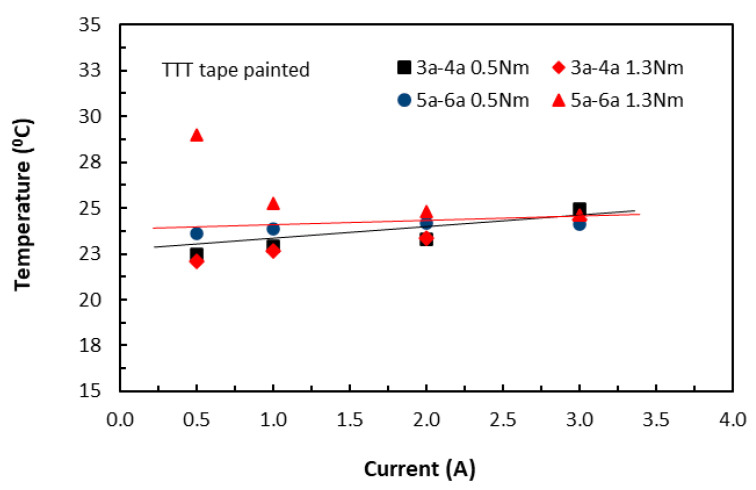
Variations of raised temperature with current on the surfaces of painted TTT specimens. (3a–4a for 20 mm length and 5a–6a for 30 mm length specimen groups. 0.5 Nm and 1.3 Nm for clamping torque levels).

In the TTT direction, the slightly different responses from both the unpainted and painted tape-based laminates were shown in Figure 6 and Figure 7, respectively. The unusually high temperature value at 0.5 A for the 30 mm long specimens (5a–6a 1.3Nm) could be due to that the aiming of a laser dot drifted towards one of the contact electrodes where temperatures were higher. They appeared to be linear, though the measured temperature variations were small. A common feature with those in the in-plane direction was that the induced temperatures were still on the rise with increase in current, even though by a small amount. Nevertheless, these findings suggested that this temperature–current relationship was independent of fibre direction, in addition to conductive paint (or fibre waviness). They further confirmed that heat transfer in the volume of carbon/epoxy laminates was influenced by epoxy resin as well as conductive fibres.

### 5.2. Relation Between Specimen Size and Raised Temperature

**Figure 8:**
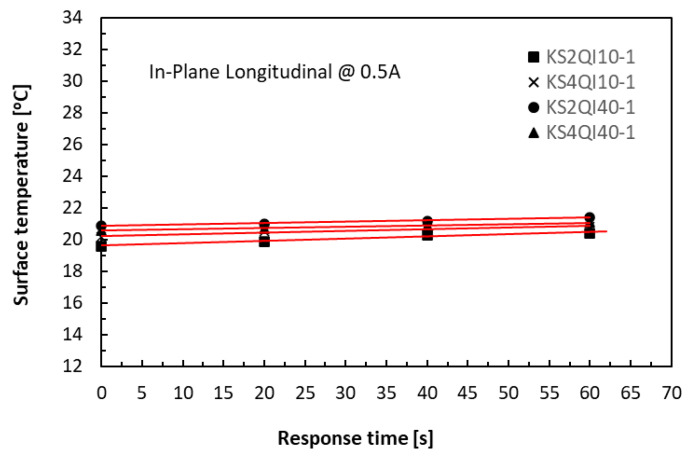
History of temperature rises in the in-plane direction of 10 mm and 40 mm long laminate specimens.

**Figure 9:**
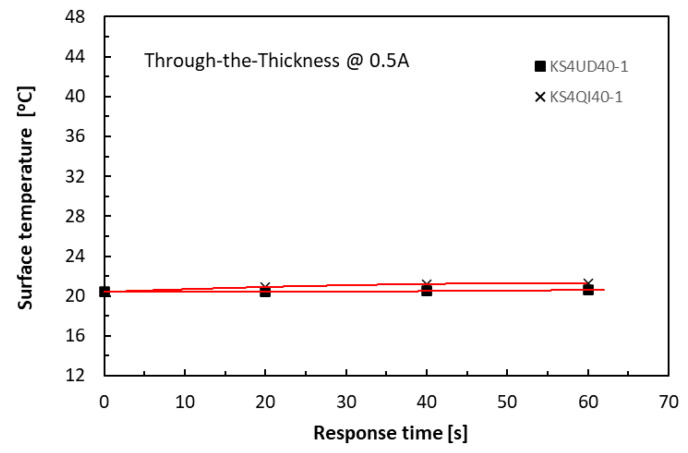
History of temperature rises in the TTT direction of 40 mm long laminate specimens.

The Joule heating-raised temperatures measured from the specimen surfaces were dependent on the volume of the specimens for the given level of supplied current, duration, and the specific heat capacity of the materials. A volume of a 40 mm long by 4 mm thick specimen is four times that of a 20 mm long by 2 mm thick one for the same given width of 10 mm. Nevertheless, the level of supplied currents was the most dominant factor. At the low levels of supplied current, say, less than 0.5 A, the joule heating was limited, and thermal equilibrium was easily achieved in both the in-plane and TTT directions. Figure 8 and Figure 9 respectively show that when the supplied level of current was low, say, 0.5 A, the effect of the specimen volumes on measured temperatures was very small for all four cases (KS2QI10 for 10 mm long 2 mm thick; KS4QI10 for 10 mm long 4 mm thick; KS2QI40 for 40 mm long 2 mm thick; and KS4QI40 for 40 mm long 4 mm thick) in both in-plane and TTT directions under any selected level of clamping torques.

**Figure 10:**
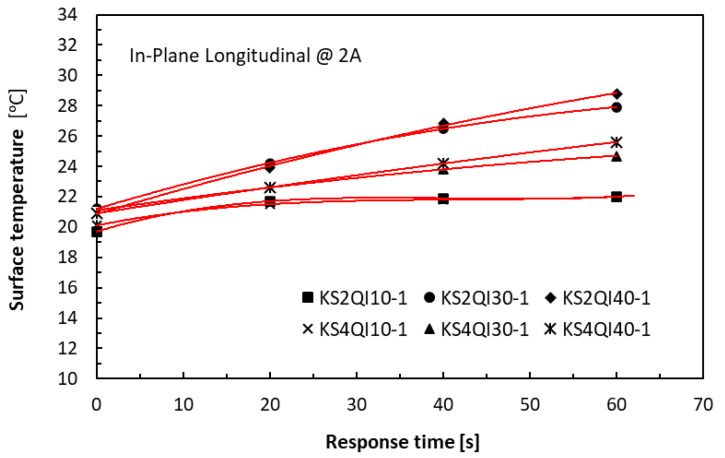
History of temperature rises in the in-plane direction of 10 mm, 30 mm, and 40 mm long laminate specimens.

**Figure 11:**
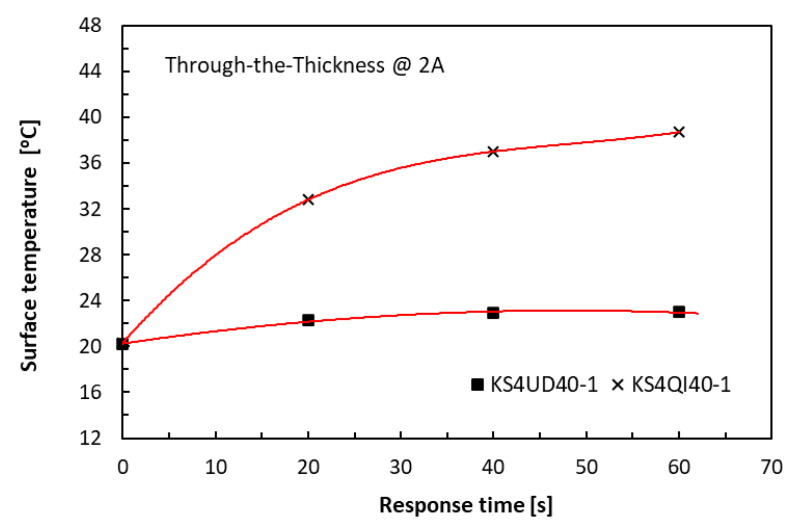
History of temperature rises in the TTT direction of 40 mm long laminate specimens.

At the relatively high current level of 2 A, the long thick specimens could accommodate much greater thermal energy within the duration of one minute than the short thin specimens without having to suffer from Joule heating damage, as similarly reported in [32]. A familiar parabolic trend of the laminate specimens in a QI lay-up could be observed in Figure 10 for the in-plane direction and in Figure 11 for the TTT direction, with the asymptote (for KS2QI10-1 group) symbolising, again, thermal equilibrium. In the latter, the lay-up effect on the measured temperatures was very significant in the TTT direction, with the UD specimens being dominated by epoxy. This was very much expected, as the temperature measurements were taken in the transverse in-plane direction, whereas carbon fibres transmitted heat effectively in the fibre direction.

### 5.3. Effect of Clamping Torques on Raised Temperature

**Figure 12:**
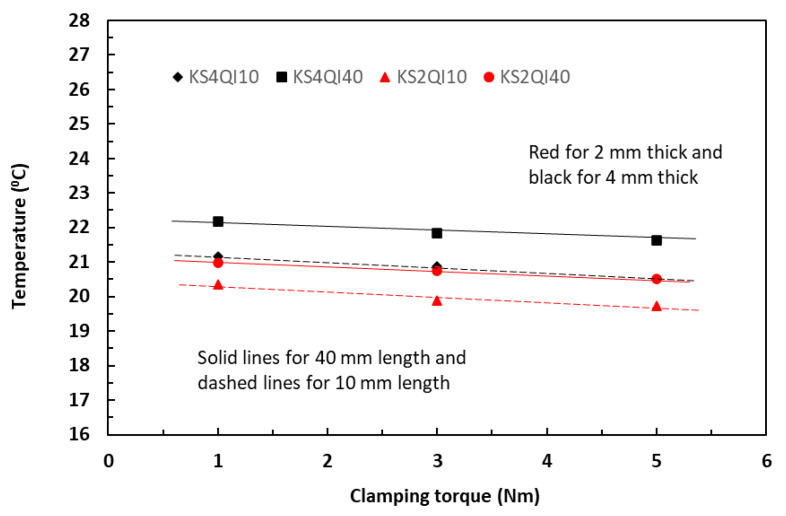
Effect of clamping torque on temperature at the current level of 0.5 A for unpainted 10 mm and 40 mm long laminates in the in-plane direction.

**Figure 13:**
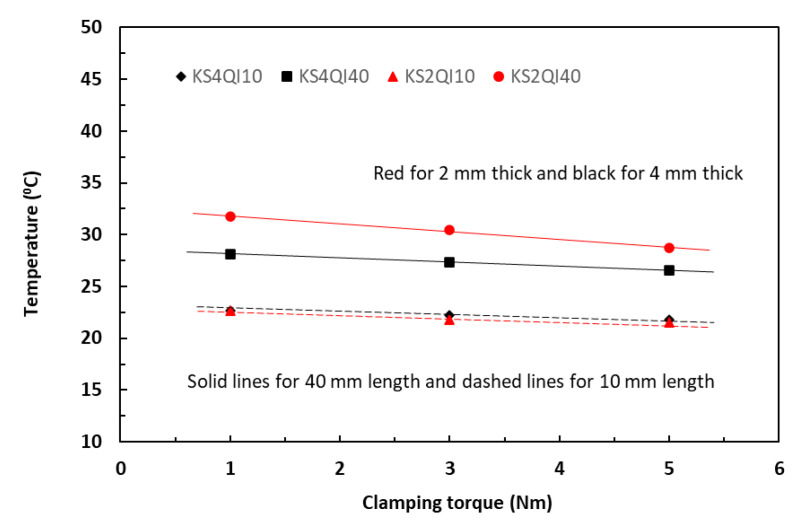
Effect of clamping torque on temperature at the current level of 2 A for unpainted 10 mm and 40 mm long laminates in the in-plane direction.

Equation (14) predicts that increase in clamping torque should result in a linear decrease in temperature. Figure 12 shows the variations of measured temperatures induced with a current level of 0.5 A with increase in clamping torque in a range of 1 to 5 Nm for the unpainted tape-based laminates in a QI lay-up. The linearly fitted data trends were consistent not only for two different thicknesses but also for two different lengths. The temperature difference among four different volumes is small, within two degrees. Figure 13 shows the similar linear temperature trends from the specimens supplied with a current level of 2 A. Moreover, it could also be observed that, across the range of the torque levels, the induced temperature differences among four different specimen volumes were much larger, in the region of eight to ten degrees, between specimens with small (200 and 400 mm^3^, dashed lines in the figure) and specimens with large volumes (800 and 1600 mm^3^, solid lines). These observations were consistent with the established in-plane data trends between current and temperature in Figure 4 and Figure 5, in which the greater levels of clamping torque led to reduction in induced temperatures.

**Figure 14:**
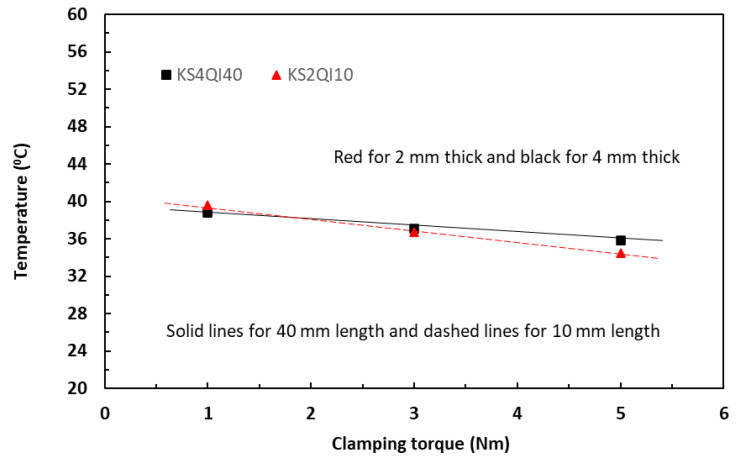
Effect of clamping torque on temperature at the current level of 2 A for unpainted 10 mm and 40 mm long laminates in the TTT direction.

In the TTT direction, the similar linear temperature trends from the specimens supplied with a current level of 2 A could also be observed in Figure 14 from the unpainted tape-based specimens with the smallest and large volumes.

### 5.4. Effect of Raised Temperature on Electrical Conductivity

**Figure 15:**
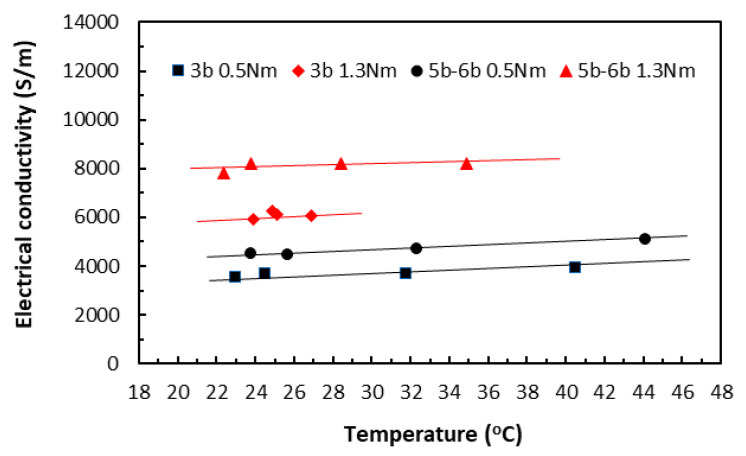
Electrical conductivities of unpainted tape-based laminates in the in-plane direction. (3b for 20 mm length and 5b–6b for 30 mm length specimen groups. 0.5 Nm and 1.3 Nm for clamping torque levels).

**Figure 16:**
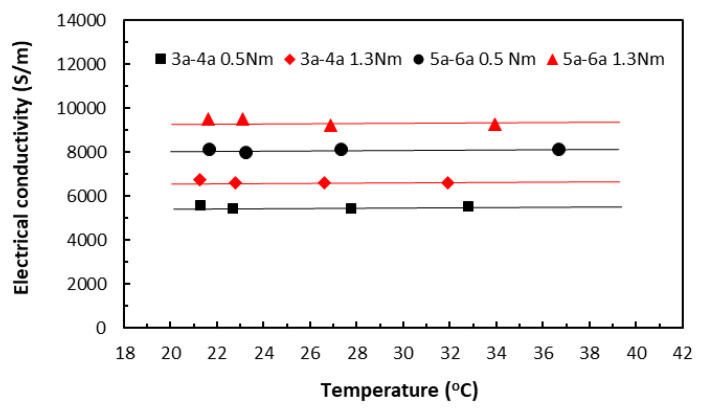
Electrical conductivities of painted tape-based laminates in the in-plane direction. (3a–4a for 20 mm length and 5a–6a for 30 mm length specimen groups. 0.5 Nm and 1.3 Nm for clamping torque levels).

When the variation of raised temperatures was well within *T_g_*, its effect on electrical conductivity could be seen in Figure 15 from the specimens of two different lengths (3bxx for 20 mm length and 5bxx for 30 mm length), each under slightly different clamping torques. Over this relatively moderate temperature range, the data trends from all the specimen groups appeared to stay nearly constant, showing little variation. Nevertheless, when all other parameters are held constant, Equation (10) predicts that the electrical conductivity–temperature relationship ought to be part of an asymptote of a specific reciprocal function, depending on the relative magnitudes of those parameters. The present data trends were thus in qualitative agreement with the prediction, without risking Joule heating damage. The very similar characteristic trends were obtained also for painted tape-based laminates in Figure 16.

**Figure 17:**
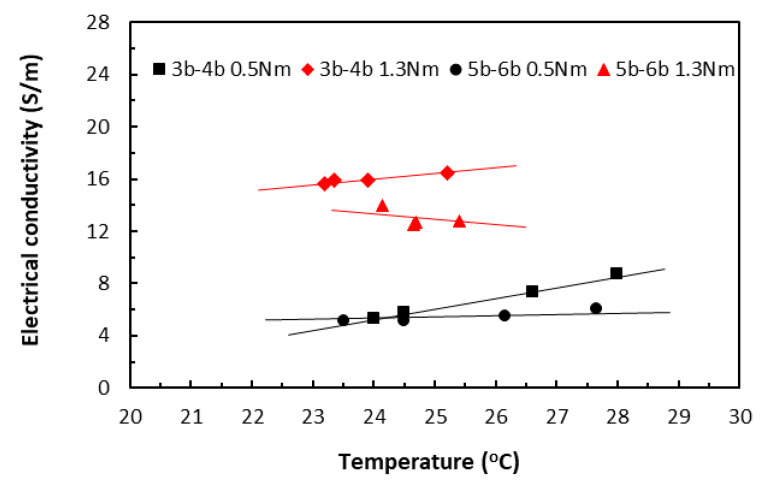
Electrical conductivities of unpainted tape-based laminates in the TTT direction. (3b–4b for 20 mm length and 5b–6b for 30 mm length specimen groups. 0.5 Nm and 1.3 Nm for clamping torque levels).

**Figure 18:**
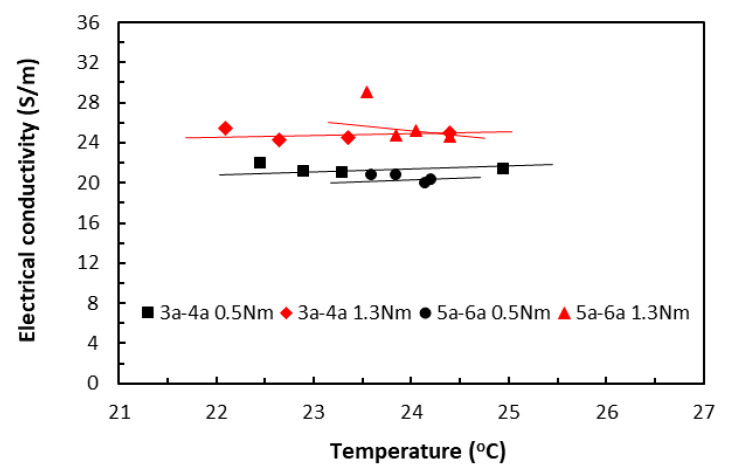
Electrical conductivities of painted tape-based laminates in the TTT direction. (3a–4a for 20 mm length and 5a–6a for 30 mm length specimen groups. 0.5 Nm and 1.3 Nm for clamping torque levels).

In the TTT direction, the thicknesses of the square specimens became the respective measurement lengths, and remained constant for these two groups of data, whereas their areas varied from 20 × 20 mm (3b–4b group) to 30 × 30 mm (5b–6b group). While Figure 17 shows the electrical conductivity data of unpainted tape-based specimens, Figure 18 shows the electrical conductivity data of the painted specimens. Again, the data trends from all the specimen groups were almost linear, showing just some small variations.

So far, the level of clamping torques showed a greater influence over the electrical conductivity through temperatures than specimen length. In all cases (except for one painted ‘3a–4a 1.3 Nm’ group in Figure 16) in both directions, data from the specimen groups under the greater clamping torque exhibited greater conductivity values. In addition, data from the painted groups in both directions exhibited greater conductivity values. This agreed with observations that the application of conductive paint improved heat transfer and dissipated some thermal energy such that the measured raised temperatures in the painted specimens were lower than those in the unpainted specimens, resulting in the greater conductivity values.

### 5.5. Anisotropic Wiedemann–Franz–Lorenz Phenomenon in Carbon/Epoxy Laminates

Joule heating induced in the measurements of electrical conductivity using the ampere-level currents raised the volume temperatures of the anisotropic laminate specimens. The Lorenz proportionality constants between the thermal conductivity *λ* (in W/mK) and the electrical conductivity *k* over the given temperature range (below *T_g_*) could be established for the same conduction mechanisms in the unpainted tape-based carbon/epoxy laminates. In the in-plane direction, the present carbon/epoxy laminates could be viewed as if they were ‘semi-conductive’ materials in terms of magnitude of electrical conductivity. Their electrical conduction mechanism was dominated by electron–phonon and electron–imperfection interactions via 61% conductive carbon fibres, whereas their thermal conduction mechanism was driven by phonon plus possibly some contribution from electron mobility [20]. In the TTT direction, the carbon/epoxy laminates could be viewed as if they were ‘barely conductive’ materials. Their electrical conduction mechanism was dominated primarily by combined elastic tunnelling-through of epoxy and phonon scattering of transverse conduction of carbon fibres, whereas their thermal conduction mechanism was dominated by phonons.

**Figure 19:**
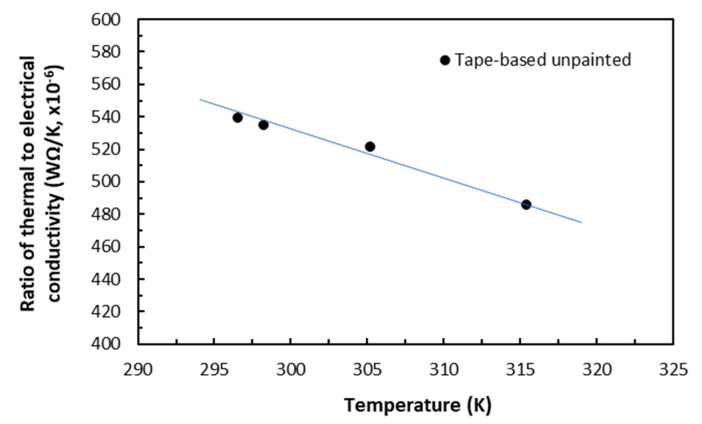
Ratio of in-plane thermal-to-electrical conductivity over temperature for unpainted laminates.

**Figure 20:**
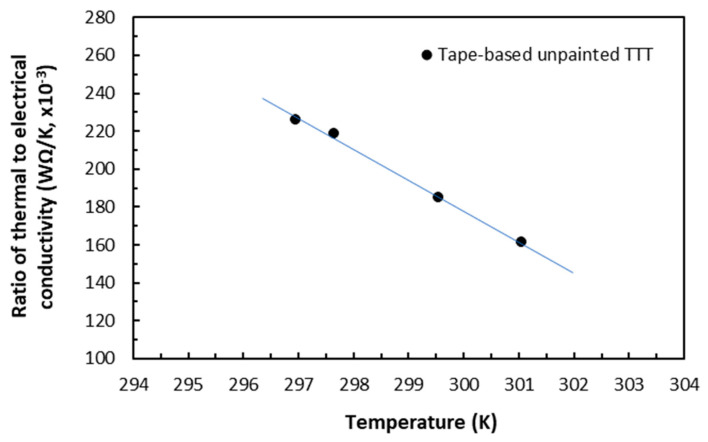
Ratio of TTT thermal-to-electrical conductivity over temperature for unpainted laminates.

As the thermal conductivity values were measured, as described in Section 4.3, without any clamping torque, for comparison, the electrical conductivity values from the same laminates in the same directions were thus extrapolated for zero clamping torque. The ratios of the thermal conductivity values (with triangle symbol) in WΩK^−2^ to the electrical conductivity values for the same temperatures in Kelvin were shown in Figure 19 and Figure 20. Figure 19 shows that in the in-plane direction, the thermal conductivity-to-electrical conductivity ratio formed a descending straight-line trend over the temperature range of 295 K (23 °C) to 317 K (43 °C). The slope of the fitted line of 32.3 × 10^−8^ WΩ/K^−2^ is the in-plane Lorenz proportionality constant *L*_L-inp_ for the present (unpainted) tape-based carbon/epoxy laminates. For metals, the typical value of Lorenz number *L_L_* is 2.45 × 10^−8^ WΩ/K^−2^. In the TTT direction, as shown in Figure 20, the Lorenz proportionality constant *L*_L-TTT_ was estimated to be 5880 × 10^−8^ WΩ/K^−2^ over the similar temperature range and this value was two orders of magnitude greater than the in-plane one. These differentiations of the magnitudes for the Lorenz proportionality constants *L*_L-inp_ and *L*_L-TTT_ were expected. This is because in metals, the conduction mechanism in heat transfer and charge mobility is dictated by electron mobility, which is the most efficient in comparison with anisotropic CFRP laminates. It was worth noting that it was slightly fortuitous for the present data points to be in a linear trend in both directions, which agreed with prediction of the WFL law over this narrow range of temperatures. There could be thermal differences between Joule heating in the electrical conduction measurements and heat-flux conduction heating in the thermal conductivity measurements before thermal equilibrium was reached.

## 6. Conclusions

The anisotropic electrical current-induced thermal behaviour of carbon/epoxy laminates were investigated using the electrical conduction measurement methodology based on 2PM via solid electrodes and the DSC thermal conduction measurements. In the former, coupon specimens in various dimensions were machined and used with or without conductive paint. The current–temperature relationships were established using induced temperatures, electrical conduction mechanisms were ascertained, and the respective effects of induced temperatures on specimen sizes, clamping torques, and electrical conductivity were examined thoroughly in both directions. In the latter, with much smaller cubic samples, the thermal conductivities of carbon/epoxy laminates were characterised using MTDSC over a wide range of temperatures. Up to *T_g_*, the thermal conductivities were found to be positively proportional to temperature increase in both directions. The analytical corroborations were developed to guide and ensure that the formed data trends were qualitatively correct, due to the pioneering nature of most experimental work and the lack of relevant results in the public domain. The Lorenz proportionality constants between thermal and electrical conductivities were established for the first time for the present carbon/epoxy laminates in a QI lay-up.

Within a glass transition temperature of the present carbon/epoxy laminates, induced temperatures were found to be moderate in most specimens of moderate and large sizes, and they formed an ascending parabolic relationship with supplied electrical current in both in-plane and TTT directions. In a few small specimens (i.e., 10 × 10 × 2 mm or 20 × 10 × 2 mm), induced rapidly rising temperatures resulted in thermal damage with the current levels of 2.5 or 3 A. Increasing clamping torques led slightly to linear reductions in temperatures. The effect of induced temperatures on the electrical conductivity was found to be very small, because the rising of temperatures did not alter the electrical conduction mechanisms. The Lorenz proportionality constants between thermal and electrical conductivities were established for the first time for these carbon/epoxy laminates in a QI lay-up. They are 32.3 × 10^−8^ WΩ/K^−2^ in the in-plane direction, and 5880 × 10^−8^ WΩ/K^−2^ in the TTT direction. This means that, in the future, for these carbon/epoxy laminates, performing either one kind of the two measurements can allow the other to be obtained.

## Data Availability

The original contributions presented in this study are included in the article. Further inquiries can be directed to the corresponding author.

## Abbreviations

The following abbreviations are used in this manuscript:

2PM	2-probe method
CFRP	Carbon fibres-reinforced plastics
CP	Cross-ply
DC	Direct current
DSC	Differential scanning calorimetry
ERTC	Electrical resistance temperature coefficient
MTDSC	Moderated temperature differential scanning calorimetry
PAN	Polyacrylonitrile
QI	Quasi-isotropic
RT	Room temperature
TGA	Thermogravimetric analysis
TTT	Through-the-thickness
UD	Uni-direction(al)
WFL	Wiedemann–Franz–Lorenz
